# Evaluating Scope and Bias of Population-Level Measles Serosurveys: A Systematized Review and Bias Assessment

**DOI:** 10.3390/vaccines12060585

**Published:** 2024-05-28

**Authors:** Alyssa N. Sbarra, Felicity T. Cutts, Han Fu, Ishu Poudyal, Dale A. Rhoda, Jonathan F. Mosser, Mark Jit

**Affiliations:** 1 Department of Infectious Disease Epidemiology, London School of Hygiene and Tropical Medicine, London WC1E 7HT, UK; 2 Institute of Health Metrics and Evaluation, University of Washington, Seattle, WA 98195, USA; 3 Department of Epidemiology, Johns Hopkins Bloomberg School of Public Health, Baltimore, MD 21205, USA; 4 Biostat Global Consulting, Worthington, OH 43085, USA; 5 Department of Health Metrics Sciences, University of Washington, Seattle, WA 98195, USA

**Keywords:** measles, seroprevalence, serology, bias

## Abstract

Background: Measles seroprevalence data have potential to be a useful tool for understanding transmission dynamics and for decision making efforts to strengthen immunization programs. In this study, we conducted a systematized review and bias assessment of all primary data on measles seroprevalence in low- and middle-income countries (as defined by World Bank 2021 income classifications) published from 1962 to 2021. Methods: On 9 March 2022, we searched PubMed for all available data. We included studies containing primary data on measles seroprevalence and excluded studies if they were clinical trials or brief reports, from only health-care workers, suspected measles cases, or only vaccinated persons. We extracted all available information on measles seroprevalence, study design, and seroassay protocol. We conducted a bias assessment based on multiple categories and classified each study as having low, moderate, severe, or critical bias. This review was registered with PROSPERO (CRD42022326075). Results: We identified 221 relevant studies across all World Health Organization regions, decades, and unique age ranges. The overall crude mean seroprevalence across all studies was 78.0% (SD: 19.3%), and the median seroprevalence was 84.0% (IQR: 72.8–91.7%). We classified 80 (36.2%) studies as having severe or critical overall bias. Studies from country-years with lower measles vaccine coverage or higher measles incidence had higher overall bias. Conclusions: While many studies have substantial underlying bias, many studies still provide some insights or data that could be used to inform modelling efforts to examine measles dynamics and programmatic decisions to reduce measles susceptibility.

## 1. Introduction

Measles remains a substantial cause of global morbidity and mortality [1], especially in low- and middle-income settings where over 99% of measles cases and deaths occur [2], despite the availability of a safe and effective vaccine [3]. Because ongoing measles transmission can be maintained if herd immunity (i.e., when the proportion of the population immune is sufficient to limit disease spread) has not been reached and sustained, estimating the proportion of people susceptible to infection within a community is essential to plan immunization programs and assess the future risk of measles outbreaks and deaths. However, due to factors such as timeliness of and age at vaccination [4], disruptions to cold chains [5], a lack of seroconversion in specific subpopulations (e.g., persons living with human immunodeficiency virus (HIV) [6]), and variable surveillance system quality across locations and time, inferring population-level measles immunity from a combination of vaccination coverage data and case notifications can be challenging [7]. Alternatively, serosurveys can provide a snapshot of immunity gaps that remain in a community by determining the population-level prevalence of IgG antibody levels above specific thresholds that suggest clinical protection against the disease.

As such, seroprevalence data can be used as tools to guide decisions to and strengthen immunization programs, as inputs to dynamic models of disease transmission, and, additionally, to provide insights into vaccine field effectiveness and for the assessment of case ascertainment rates [7,8]. The interpretation of seroprevalence data is complicated, however, because of the potential for bias. Some of this bias can be due to inadequate sensitivity of the laboratory assays [9] and/or specimen types [10] used for measuring antibody levels. Additionally, bias from assay procedures can be suspected when protocols or commercial details are not reported or if no quality control was performed. Furthermore, population-based surveys have the potential for additional bias to be introduced in the selection of the participants or from lack of representativeness of the selected sample from the community.

Beyond understanding the selection processes and laboratory assays used, it is critical to also consider how the results of serosurveys are reported. Considerations include what threshold of antibody titer was used as a correlate of clinical protection and how some tests report indeterminate results. In order to responsibly use and accurately interpret seroprevalence data for decision making or for modelling exercises, these issues need to be transparently acknowledged and discussed.

A more in-depth understanding of the available seroprevalence data across locations and time, as well as the related implications, is critical for using these historic data to calibrate models used to inform decision making for immunization program strengthening, especially in low- and middle-income countries (LMICs) that face the highest ongoing measles burden. To fill these gaps, we first conducted a systematized review of the literature reporting measles seroprevalence data published through 2021 and extracted information on key study and assay information. Then, we developed a pilot bias assessment tool to assess the risk of bias in each study across the following categories: study selection of the participants, measurement tool and classification of immunity, and results reporting.

## 2. Materials and Methods

### 2.1. Search Strategy and Selection Criteria

This study followed PRISMA guidelines (Supplementary Tables S1 and S2) and was registered with PROSPERO (CRD42022326075). We performed a systematized review of the published literature in any language containing information on population-level measles seroprevalence in LMICs. We searched PubMed on 9 March 2022 for primary data published through 31 December 2021 using the following search string:

(((Measles) AND (seroprevalence OR sero-prevalence OR seropositive OR sero-positive OR seronegative OR sero-negative OR seroepidemiology OR sero-epidemiology OR seroprofile OR seroimmunity OR sero-immunity))

OR (“Measles/epidemiology”[MeSH] AND (antibod* OR serolog*)))

AND (“1900”[Date—Publication]: “2021”[Date—Publication])

One individual (ANS) screened titles and abstracts for each study in the search results. For relevant studies, one of multiple individuals (ANS, HF, IP) reviewed the full text of each to determine their inclusion or exclusion. We included studies that contained original data on measles antibody prevalence and excluded studies if they only contained data from high-income locations (based on WorldBank 2021 income classifications [11]), did not contain data on measles IgG antibodies, were based on non-original data or were from non-human subjects, contained only results from laboratory assay development or clinical trials (including studies only containing information on vaccinated persons), studied a target population of only health-care workers or active measles cases, or were a review, abstract, letter, editorial, or brief report.

Following the full-text review, for each study that met our inclusion and exclusion criteria, we extracted the following data: study setting, study design and type (including information on planned, achieved (i.e., how many persons were reached via sampling), and reported (i.e., how many persons were represented in the final study metrics) sample sizes, population demographics (including income level, urban or rural, and representativeness), type of specimen collected, serologic assay details (including type, name, and inclusion of a reference preparation), antibody threshold used for seropositivity and/or seroprotection (if relevant), and measures of proportion seropositive, seronegative, or indeterminate with accompanying uncertainty. The studies used varying quantitative thresholds to define seropositive, seronegative, or indeterminate or equivocal results. In this analysis, we extracted and present these metrics as reported by each individual study, while also extracting the specific antibody thresholds for seropositivity used where available. We extracted the data into a Microsoft Excel workbook (Microsoft 365) and, for the seroprevalence measure, we recorded the most granular levels for the relevant strata (i.e., by age, subnational geography, vaccination status, infection history, etc.) presented in each study.

### 2.2. Bias Assessment

Following the extraction of all available data, we developed a comprehensive bias assessment tool and applied the tool to characterize the level of bias across each study. Our tool, modified from the ROBINS-I tool [12], considers bias across the following categories, with associated indicators: study selection of the participants, measurement tool and classification of immunity, and reporting of the results (Supplementary Figures S1–S3). We classified the level of bias across each category to be either low, moderate, severe, or critical. We then finally assessed the overall level of bias as low, moderate, severe, or critical for each study by taking the mean score of the category-specific classifications.

To assess bias in the study selection of the participants, we considered whether the study design used a random process for sample selection, if a study relied on a convenience sample, was restricted to only a subset of the population (e.g., only included pregnant women or cancer survivors), and reported the planned, achieved, specimen, and final sample sizes. To assess the level of bias in the measurement tool and classification of immunity, we considered whether assay protocol, name, or references were provided, if internal or external validation or quality control was performed, and if there were other known factors known to decrease sensitivity or specificity (i.e., using oral fluid samples as specimens [13], using a hemagglutination inhibition (HI/HAI) assay [13], or using the Whittaker enzyme-linked immunosorbent assay (ELISA) [14]). Last, for bias in the reporting of the results, we considered whether a known threshold (i.e., included in the published literature) was used for determining the protective titer levels, including the metrics of uncertainty with seroprevalence estimates, and, if an enzyme immunoassay (EIA) or ELISA was used, whether and how equivocal results were handled and reported.

We characterized the overall level of bias in each study using the following criteria. For each category of bias, the studies were given a numeric score: low bias was assigned a score of 1, moderate bias a score of 2, severe bias a score of 3, and critical bias a score of 4. We took the sum of the scores across all three categories. Studies with a score sum from 3 to 4 were characterized to have low overall bias, those with a score sum from 5 to 7 to have moderate overall bias, those with a score sum from 8 to 9 to have severe bias, and those with a score sum from 10 to 12 to have critical bias.

We converted all metrics reported to proportion seropositive and then used R version 5.4.0 to compute the summary metrics and create the figures. For studies reporting seropositive and indeterminate/equivocal results independently, we did not include indeterminate results in the numerator of our overall seroprevalence calculation. We compared data availability by decade and bias level. We additionally investigated the bias levels across time and region and assessed the bias levels across locations with higher and lower first-dose measles-containing vaccine (MCV1) coverage (as reported by WUENIC [15]) and higher and lower estimated annual measles incidence (as estimated by a state–space model and described elsewhere [16]) in the year from which the study data were collected. To examine the relationship between MCV1 coverage and overall bias as well as annual measles incidence and overall bias, we used separate proportional odds logistic regression models for each coverage and incidence and assessed the coefficient significance.

## 3. Results

### 3.1. Systematized Review

**Figure 1:**
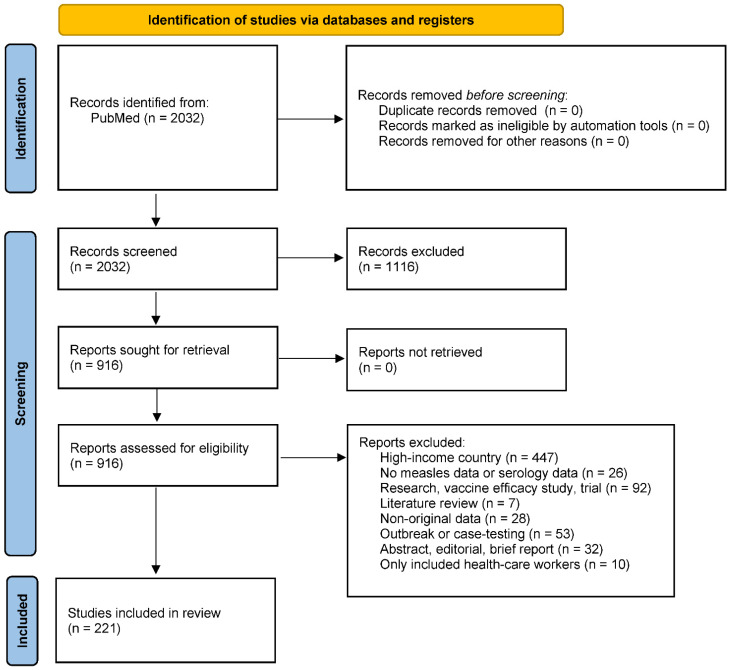
PRISMA diagram.

From our search, we identified 2032 studies for screening (Figure 1). Following screening, we excluded 1116 studies that did not meet our search criteria. For the remaining 916 studies, we assessed the full-text articles for inclusion. We identified 221 studies for inclusion and extracted information on measles seroprevalence, study design, and seroassay (extracted data by age, geography, vaccination or infection status as available) can be downloaded from: https://ghdx.healthdata.org/record/ihme-data/measles-serosurvey-bias-systematic-review (accessed on 24 May 2024). The studies were published between 1962 and 2021, including seroprevalence surveys conducted between 1953 and 2019.

Among the 182,789 persons sampled across all studies, age groups, and years, the crude mean measles seroprevalence was 78.0% (SD: 19.3%), and the median seroprevalence was 84.0% (IQR: 72.8–91.7%). Among the studies reporting results stratified by sex, the crude mean measles seroprevalence was 82.2% (SD: 17.8%) among females (n = 36,884) and 79.1% (SD: 19.1%) among males (n = 32,720), and the median seroprevalence was 87.5% (IQR: 80.8–93.1%) among females and 86.6% (IQR: 72.9–91.5%) among males.

**Figure 2:**
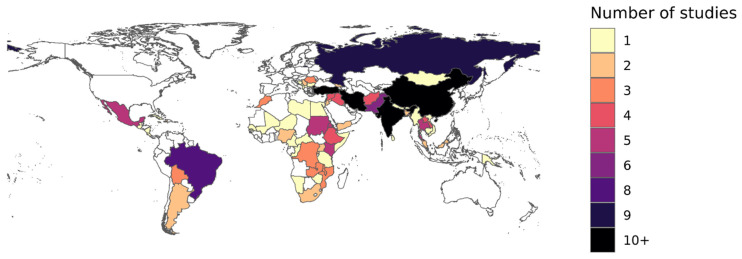
Number of serosurveys with data included per country. Map of the number of studies per country with available data identified by the systematized review.

Across regions of the World Health Organization (WHO), there were 43 studies containing data from the African Region, 47 from the Eastern Mediterranean Region, 35 from the European Region, 25 from the Region of the Americas, 20 from the South-East Asia Region, and 73 from the Western Pacific Region (Figure 2). There were 24 studies that represented data collected before 1980, 32 studies with data from 1980 to 1989, 29 studies with data from 1990 to 1999, 55 studies with data from 2000 to 2009, and 83 studies with data from 2010 to 2019. In addition, 178 studies (80.5%) contained age-stratified results across 531 unique age ranges.

### 3.2. Bias Assessment

Table 1 shows the results of our bias assessment for each included study. For overall bias, we classified bias as low in 12 (5.4%) studies, moderate in 129 (58.3%), severe in 58 (26.2%), and critical in 22 (10.0%). No studies had low or critical bias across all the categories of study selection of the participants, measurement tool and classification of immunity, and reporting of the results (Table 1).

For study selection of the participants, we identified 15 studies with low bias, 181 with moderate bias, 23 with severe bias, and 2 with critical bias. In addition, 81 studies used a random sample selection method, 117 studies with convenience samples used a restricted, non-representative sample (i.e., only including a specific subgroup of the population, such as persons living with HIV), 2 studies did not report the final sample size, and of the 81 samples that used a random sample selection method, 45 reported the planned sample size, and 15 additionally reported the planned, achieved, and specimen sample sizes.

For measurement assay and classification of immunity, we identified 19 studies with low bias, 130 with moderate bias, 46 with severe bias, and 26 with critical bias. Across the three categories of bias assessment, measurement assay and classification of immunity had the highest number of studies classified as having critical bias, largely due to the absence of information on assay protocol details, commercial kit name, or other appropriate citation describing the underlying methods. There were 195 studies that provided details on the assay protocol or commercial kit name, and 25 studies that conducted internal or external validation or quality control. Also, 6 studies specified that the samples were oral fluid specimens, and 30 studies specified that the samples collected were dried blood spots.

We also found that 54 studies used an HI/HAI assay, 139 used an EIA or ELISA, 13 used a plaque reduction neutralization test (PRNT), 6 used a multiplex bead assay, and 11 used other or undescribed assay types. We noted changing temporal trends of the types of seroassays used. While EIA, ELISA, and PRNT assays were used in even distribution across all studies examined, there was no study published after 2001 that utilized an HI/HAI assay, and all studies using a multiplex immunofluorescent assay were conducted in 2013 or later.

We identified 20 studies with low bias, 63 with moderate bias, 70 with severe bias, and 18 with critical bias in reporting of the results. One hundred fifty-five studies reported a threshold to define seroprevalence. Among the 139 studies that used an EIA or ELISA, 30 studies reported equivocal results separately or included with seropositivity results, and 1 study excluded equivocal results; they were less than 5% of the overall sample. Finally, 59 studies reported metrics of seropositivity or seronegativity with any accompanying uncertainty.

### 3.3. Seroprevalence Trends

**Figure 3:**
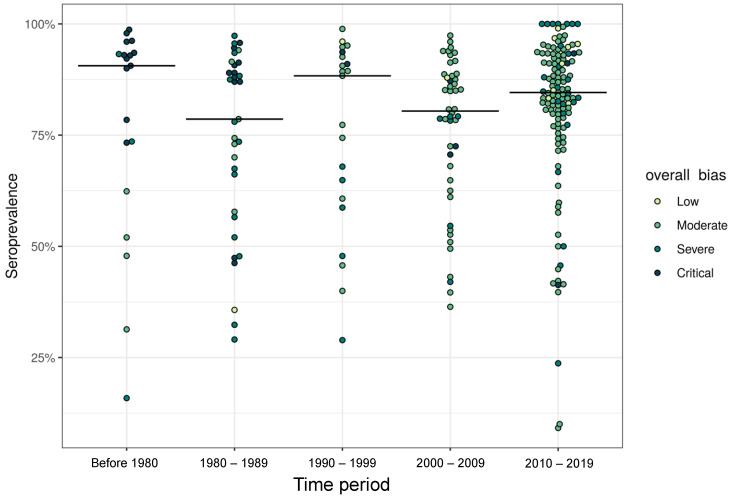
Measles seroprevalence by time period and overall bias level. Beeswarm plot of measles seroprevalence by time period. Each point represents one country-year of data per study and is colored according to the overall bias level. The black lines represent the median observation across each decade.

The crude median seroprevalence estimate from the studies in the Western Pacific Region was 88.3% (IQR: 79.2–93.4%), from those in the Eastern Mediterranean Region was 87.2% (IQR: 81.3–93.2%), from those in the European Region was 82.0% (IQR: 77.8–89.0%), from those in the Region of the Americas was 78.4% (IQR: 60.7–93.0%), from those in the African Region was 77.6% (IQR: 60.7–89.9%), and from those in the South-East Asia Region was 66.8% (IQR: 47.4–88.4%). The trends in seroprevalence and bias varied by decade (Figure 3). The median seroprevalence was lower in studies from 2010 to 2019 than in those conducted before 1980 (i.e., in the pre-vaccination era). The crude seroprevalence from studies conducted before 1980 was 90.5% (IQR: 67.8–93.3%), that from 1980 to 1989 was 78.6% (IQR: 57.8–90.7%), that from 1990 to 1999 was 88.3% (IQR: 60.7–92.6%), that from 2000 to 2010 was 80.4% (IQR: 65.6–88.2%), and that from 2010 to 2019 was 84.6% (IQR: 78.3–92.9%). Of the 31 country-years with studies containing critical bias, 23 (74%) occurred in earlier time periods (i.e., before 1980 and between 1980 and 1989). Of the 159 country-years with studies containing low or moderate bias, 96 (60%) occurred between 2010 and 2019.

**Figure 4:**
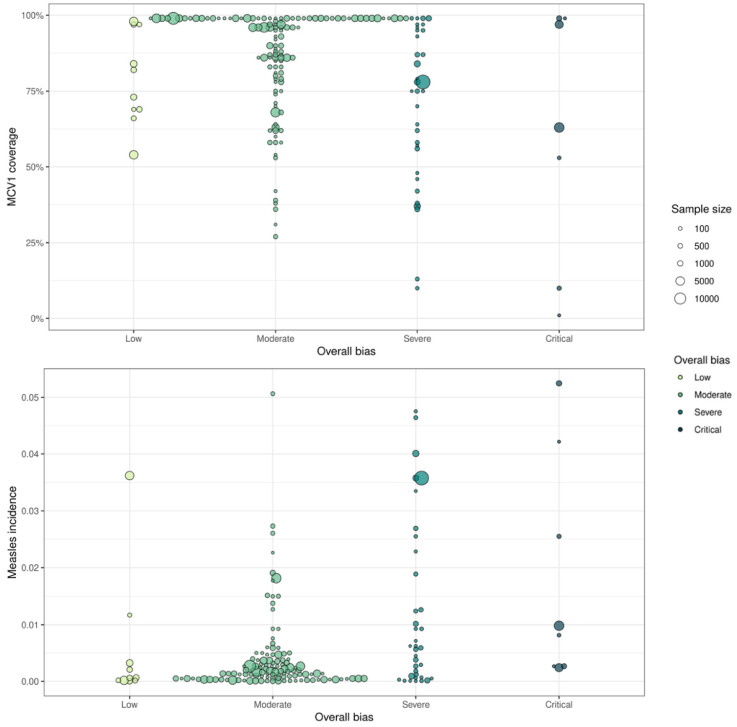
Overall bias level by MCV1 coverage and annual measles incidence. Each point represents each country-year across all studies, the overall bias level by MCV1 coverage (**top**), and the annual estimated measles incidence (**bottom**).

We additionally compared the overall bias levels for each country-year of the studies to the MCV1 coverage and measles incidence from the same country-year (Figure 4). Generally, the studies in countries and years (1980 or later) with lower MCV1 coverage and higher measles incidence had more bias compared to the studies from countries and years with higher MCV1 coverage and lower measles incidence (*p* < 0.001, in proportional odds logistic regression models for both MCV1 coverage and incidence). Among the 109 studies from countries and years with MCV1 coverage greater than 80%, 93 (85%) had low or moderate overall bias, and among the 58 studies from countries and years with MCV1 coverage of 80% or lower, 34 (58%) had low or moderate overall bias. A similar trend persisted across studies in countries and years with high incidence: 103 of the 122 (84%) studies in countries and years with average annual reported measles incidence less than 5 per 1000 persons had low or moderate overall bias, and 24 of the 49 (49%) studies in countries with annual measles incidence of 5 per 1000 persons or greater had low or moderate overall bias.

## 4. Discussion

To identify the scope of measles seroprevalence data, we conducted a systematized review of serosurveys to identify primary data sources and characterized the underlying bias across these studies. The resulting data repository from our investigation along with information on factors related to the underlying bias per study could contribute to analyses of measles dynamics among low- and middle-income countries. We identified serosurveys available in each decade, WHO region, and across a wide variety of ages, which could be useful when modelling location-, time-, and age-specific estimates of measles transmission and susceptibility. Despite this variation, there were locations for which very few or no serosurveys have been conducted—mainly in the African Region—which contribute to knowledge and data gaps to inform high-quality modelling and analyses.

Two previous reviews were conducted on measles seroprevalence. The first by Thompson and Odahowski [17] summarized the available serological data on immunizing antibodies (i.e., IgG) against measles and rubella through June 2014. The systematic review found 72 countries with data from at least one study. An additional review by Dimech and Mulders [18] included studies from 1998 to 2014. In this, 68 articles containing measles seroprevalence data were included. The review contains information on measles seroprevalence, along with some details on study population, sample size, and measurement assay. Both reviews, however, only contain data from a limited time window, do not provide critical information required from a thorough review, and did not go on to assess each study by its underlying characteristics.

In addition to expanding the time window searched and the depth of information provided, our study provides insight to issues to consider when designing and reporting a seroprevalence study to ensure that the highest quality surveys are conducted and that complete, accurate, and transparent reports are generated. The number of available measles seroprevalence studies has increased in the last few decades compared to periods before the introduction of national measles vaccination programs in LMICs. This trend provides the opportunity for researchers to examine the impact of vaccination programs on ongoing susceptibility within the population represented in each study. However, we found that locations with high annual measles incidence and lower MCV1 coverage tend to have not only less studies conducted, but also higher bias—this is understandable, given that coverage tends to be lower in the most difficult settings such as remote and/or conflict-affected regions, where surveys are especially challenging to conduct. Research and programmatic teams planning seroprevalence studies, especially among persons living in these vulnerable communities, could use the framework presented in this study as a starting point for determining the feasibility and cost of conducting a high-quality seroprevalence survey and consider alternative ways to invest funds (e.g., in strengthening the ongoing surveillance of coverage and disease incidence).

More recently, there have been examples of high-quality serosurveys, such as a nationally representative survey in Zambia [19], that have been conducted and used for informative modelling. Given the complexity, time, and expense of these surveys, it is worthwhile to make the most of high-quality surveys that are being conducted for different infections and funded through a variety of different programs. This serosurvey in Zambia, for example, leveraged residual sera from the Zambia Population-Based HIV Impact Assessment (ZAMPHIA) study [20] originally collected to estimate HIV incidence and viral load. Applications of such data extend to innovative modelling efforts to determine subnational and age-specific seroprevalence estimates as well as national-level outbreak risk [19]. That study serves as an example of the potential to leverage other major population surveys and to use high-quality seroprevalence estimates to inform evidence for decision making.

More studies had low or moderate bias than severe or critical bias for the categories of selection of the study participants and measurement tool and classification of immunity. For the category of reporting of the results, more studies had severe or critical bias levels than low or moderate bias levels. Overall, we found that less than 10% of the studies had low overall bias, suggesting that the quality of conduct and reporting of seroprevalence studies has substantial potential for improvement.

While interpreting seroprevalence estimates identified by our review, it is essential to also consider the associated sensitivity and specificity of the seroassays used in the studies, along with the route of induced immunity (i.e., from vaccination or natural infection). For example, HI/HAI assays are often less sensitive than other types of assay [13]. If HI/HAI assays are used in a population with mainly vaccine-induced immunity, seroprevalence may be underestimated. However, since HI/HAI assays were historically used more frequently, during an era with less vaccine-derived immunity and subsequently higher natural immunity affording higher antibody levels, assay sensitivity might not be as important to consider. In our bias assessment in the category of measurement tool and classification of immunity, we defined the factors that influence assay specificity and sensitivity as (1) using an HI/HAI assay, (2) using the Whittaker commercial ELISA kit, (3) using oral fluid samples. However, the utility of this specific contribution to our bias assessment might be subject to the specific study setting, vaccination program implementation and success, and underlying measles epidemiology.

In this study, we developed a framework to categorize the risk for bias (i.e., a systematic deviation) among seroprevalence estimates. Characterizing bias is important because it can impact estimate interpretability. However, this study did not attempt to directly quantify or characterize uncertainty (i.e., the degree to which a result is known) in the seroprevalence estimates. While sources of uncertainty can be challenging to characterize overall, similar factors that contributed to our assessment of bias could also impact the underlying uncertainty of the seroprevalence estimates identified in this study. These include uncertainty resulting from the test results themselves (e.g., measurement uncertainty from the assays) as well as uncertainty resulting from the sampling process or survey design. While studies that are highly biased might also have high uncertainty, there are other factors not considered in this review that might change this relationship. For example, a well-designed study with a small sample size may have a low risk of bias, yet high uncertainty. In data extraction and when assessing the bias in the reporting of the results category, we did note whether studies reported metrics of uncertainty along with seroprevalence measures. We did not, however, consider the width of these ranges in assessing the bias levels.

While serologic technology has largely improved over the last 60 years, there is still utility in understanding what literature is available from all available years. First, examining historical data allows us to investigate measles seroprevalence before the widescale expansion of vaccination programs and to better understand measles transmission dynamics in the absence of immunization programs. Additionally, as plaque reduction neutralization was developed in the 1980s, data from previous decades may still be of high quality. Finally, a major use case of these data includes the calibration of dynamic models of measles transmission; having access to data to calibrate or validate a model for as many years as possible, with a proper understanding of possible biases that might be related to each study that we have suggested in this work, is an advantage and adds additional resources to modeling toolboxes.

Our study has several limitations. First, we were unable to fully synthesize the results of our systematized review in a meta-analysis or other stratified analysis by age, location, or year or present other summarized metrics. This was due to the level of bias present in much of the literature, underlying the changing vaccination coverage across our study period, the heterogeneity across settings, and the relative data sparseness by country across time. Given the bias in the serology data, the appropriate way to understand historical measles dynamics in these countries would be to combine and/or triangulate serology with other data sources (e.g., vaccination coverage, measles incidence, outbreak frequency, population demography). The results presented in this study can serve as the basis for future models that synthesize data while also accounting for underlying measles infection dynamics, vaccination coverage, and population structures for each individual study setting, which was out of the scope of our analysis.

Secondly, we were constrained by the information reported in each publication. Without adequate reporting, we assumed the highest level of associated bias whenever appropriate. For example, if a study did not specifically note if an international reference preparation was used, we assumed that it was not used. This may have led us to classify studies as having a higher bias in relevant categories than might have been the case if all available information had been included in the publication—it is possible that some details were omitted to meet restrictions on word counts, for example. As such, there might be great utility in the widespread use of standardized reporting expectations for ongoing and future seroprevalence studies.

Next, we did not consider the sample size in our assessment of bias. Since the impact of the sample size on the reliability of point estimates from seroprevalence studies should be reflected in the provided uncertainty interval, we considered the inclusion of such in our bias assessment. We did not, however, further assess the implications of smaller or wide interval spans if they were presented or whether point estimates or uncertainty intervals were adjusted or standardized for population demographics or other factors. Additionally, there are likely additional sources of bias that are more difficult to ascertain objectively, such as potential issues with specimen storage and laboratory capacity, practices, and quality. Finally, we only included studies from LMICs in our study, as they are locations with the highest measles burden. Future studies investigating measles seroprevalence in high-income countries, especially among localized populations or networks, are recommended.

Our study strengthens the understanding of the availability of and bias among measles seroprevalence studies in low- and middle-income countries by identifying primary sources of measles seroprevalence data and conducting a bias assessment of the associated data. Our framework for assessing bias could provide a foundation for further work by relevant agencies and interested partners to develop a tool for use in planning and reporting future surveys. This work can be a vital tool to be used during modelling exercises, planning immunization-based interventions, and ultimately, to make informed decisions to reduce preventable measles morbidity and mortality.

## Figures and Tables

**Table 1:** Overall and categorical bias classifications. Results of bias assessment in each of three categories (study selection of the participants, measurement tool and classification of immunity, and reporting of the results) and overall level of bias per study.

Level of Bias	Study Selection of Participants	Measurement Tool and Classification of Immunity	Reporting of Results	Overall
Low	15	19	20	12
Moderate	181	130	63	129
Severe	23	46	70	58
Critical	2	26	18	22

## Data Availability

The data presented in this study are available in this article and Supplementary Materials.

## Supplementary Materials

The following supporting information can be downloaded at: https://www.mdpi.com/article/10.3390/vaccines12060585/s1, Figure S1: Bias of selection of study participants flowchart; Figure S2: Bias of measurement tool and classification of immunity flowchart; Figure S3: Bias of reporting of results flowchart; Table S1: PRISMA abstract checklist; Table S2: PRISMA checklist.
